# The Antisecretory Factor in Plasma and Breast Milk in Breastfeeding Mothers—A Prospective Cohort Study in Sweden

**DOI:** 10.3390/nu10091227

**Published:** 2018-09-04

**Authors:** Anna Gustafsson, Elisabeth Granström, Christina Stecksén-Blicks, Christina E. West, Sven-Arne Silfverdal

**Affiliations:** 1 Department of Clinical Science, Intervention and Technology, Karolinska Institutet, SE 141 86 Stockholm, Sweden; 2 Department of Neonatology, Karolinska University Hospital, SE 171 76 Stockholm, Sweden; 3 Department of Odontology, Pediatric dentistry, Umeå university, SE 901 87 Umeå, Sweden; elisabeth.granstrom@umu.se (E.G.); christina.stecksen-blicks@umu.se (C.S.-B.); 4 Department of Clinical Sciences, Pediatrics, Umeå University, SE 901 87 Umeå, Sweden; christina.west@umu.se (C.E.W.); sven.arne.silfverdal@umu.se (S.-A.S.)

**Keywords:** antisecretory factor, human milk, breast milk, breastfeeding, inflammation, lactoferrin, candida

## Abstract

Inflammation and infection postpartum threaten the mother and her infant. Human milk provides a defense for the infant, but inflammatory complications like mastitis may lead to the cessation of breastfeeding. Antisecretory factor (AF) has a role in the regulation of secretory processes and inflammation. The objective of the study was to describe AF-levels in plasma and breast milk, and in relation to breast complications. Breastfeeding mothers (*n* = 95) were consecutively recruited at a Well Baby Clinic in Umeå, Sweden. At inclusion four weeks postpartum, samples of venous blood (10 mL) and breast milk (10 mL) were collected. Active AF was analyzed with ELISA using a monoclonal antibody mAb43, and was detected in all samples of plasma and breast milk with a positive correlation (Spearman coefficient = 0.40, *p* < 0.001; Pearson correlation = 0.34, *p* < 0.01). High AF-levels in plasma correlated with high AF-levels in breast milk. The results suggest a co-regulation between active AF in plasma and breastmilk, and/or a local regulation of AF in the breast. Further studies are needed to determine the pathways for the activation of AF-levels in breast milk and plasma.

## 1. Introduction

Infection and inflammation constitute a threat to the newborn infant and its mother. The gut of the human newborn infant is susceptible to inflammation and inflammatory conditions that may affect the growth of the infant and cause complications, especially in infants born preterm [1,2]. Human milk provides a broad anti-inflammatory defense [3], but sometimes lactation is threatened by inflammatory complications like mastitis that can lead to the cessation of breastfeeding [4]. Mastitis in lactating women is defined by the World Health Organization (WHO) as an inflammatory condition of the breast, which may or may not be accompanied by infection [5]. It varies in severity, ranging from mild symptoms with local inflammation in the affected breast to more serious symptoms including fever, abscess, and septicemia, requiring interventions [6] that may lead to the cessation of breastfeeding [4]. Recent studies have suggested that inflammation in subclinical mastitis may cause a low milk supply and hence increase the risk of impaired growth of the infant, as well as the cessation of breastfeeding [7]. 

Human milk contains a variety of biologically active components and is involved in the development of the infant immune system and intestinal microbiota [8,9]. The bioactivity of breast milk has been shown to influence gene expression in the neonatal gastrointestinal tract, with a different expression in formula-fed infants when compared with breast-fed infants [10]. Secretory IgA antibodies bind the microbes on the infant’s mucosal membranes, preventing activation of the pro-inflammatory defense. Lactoferrin, a major milk protein, is an important protein for protection against inflammation and disease and reduces inflammatory responses. Additionally, the non-absorbed human milk oligosaccharides block the attachment of microbes to the infant’s mucosa, preventing infectious diseases [11]. Transfer of numerous cytokines and growth factors via milk may also activate the infant’s immune system. Recent studies have detected a human milk microbiome [12], which is suggested to be influenced by factors like mode of delivery, duration of breastfeeding, and place of habitation.

Antisecretory factor (AF) is a protein which regulates secretory processes and inflammation and that might be of importance not only in animals, but also in infants in the postnatal period [13]. AF is present in most human tissues and body fluids, as well as in the placenta and in breast milk [14,15], with a suggested role in the immune system due to expression on macrophages, B-cells, and dendritic cells, and in all secondary lymphoid organs [16]. A high expression of AF is restricted to specific cell populations, such as certain types of epithelia, neuron, endocrine cells, and subgroups of leukocytes [13]. Cells that store AF also have the capacity to synthesize AF. It probably exerts its effects via nerves, but other mechanisms via receptors, binding proteins, and transport channels in the cellular membrane might be involved. AF can be detected in plasma, and is mostly present in an inactive form in healthy persons [13]. Active endogenous AF in plasma increases by exposure to enterotoxins and certain food constituents. An enhanced activation of endogenous AF synthesis improves the clinical outcome in diseases characterized by inflammation and secretory dysfunction in both humans and animals [13,14,17]. In animals, the levels of active AF in breast milk are positively correlated to the levels in plasma, with a higher concentration in milk than in plasma, probably due to the active transport of active AF across the epithelial lining of the mammary gland [13]. Studies of animals have demonstrated that levels of active AF in plasma and milk can be enhanced through an AF inducing diet, with a protective effect in the offspring related to growth and health [18].

In humans, an intervention study in breastfeeding mothers showed that the induction of endogenous AF in breast milk with specially processed cereals (SPC-flakes^®^) prevented mastitis [15]. An increased AF plasma level has so far not been reported to induce any form of medical side effects [19].

There is, to our knowledge, no previous descriptive study on the basic levels of active AF in plasma and breast milk in breastfeeding women. An increased knowledge of the basic levels of AF in plasma and breastmilk, and their relation, may be a base for intervention studies, aiming to prevent inflammatory complications in both the mother and infant and promote breastfeeding, as well as infant growth and health. The objective of the study was to describe the basic levels of AF in plasma and breast milk in a cohort of breastfeeding mothers in Sweden.

## 2. Materials and Methods

### 2.1. Study Population and Ethical Approvals 

This study was prospective, explorative, and descriptive, and a sub-study of an investigation of oral candida infection in infants [20] with 100 mother-infant pairs included. The levels of the outcome parameters, candida colonization in newborns, and antisecretory factor in the mothers were not known. We estimated that in this exploratory study, 100 children needed to be included to obtain an acceptable accuracy. A total of 120 mothers and their four-week old infants were invited to participate in a consecutive order at a single Well Baby Clinic in Umeå, Northern Sweden, between April 2011 and September 2012, of whom 20 did not consent to participate. Samples from plasma and breast milk were collected from 95 of the mothers. The study was approved by the Regional Ethical Review Board in Umeå, Sweden (Dnr 2010-218-31M and 2014-341-32M). Before entry, both parents signed written consent.

### 2.2. Questionnaire

At inclusion, mothers were asked to fill in a questionnaire about dietary habits on the consumption of an AF-inducing diet consisting of SPC-flakes^®^ and other cereals on the market (i.e., breakfast cereals, grain, porridge) that also might induce/activate AF. Questions were also asked about living conditions, delivery mode, infant weight and length at birth, infant feeding, smoking in the family, and use of antibiotics in the mother and infant. Data on the mother’s BMI (kg/m^2^) were calculated using data on weight and length collected from charts at the last visit during pregnancy at the maternity clinic, and data were available for 86 mothers. At 12 months postpartum, the mother was asked about a history of maternal infections and inflammation, including mastitis and sore nipples.

### 2.3. Samplings and Analysis of Antisecretory Factor (AF), Lactoferrin and Calcium 

From the mothers, samples of venous blood (10 mL) and breast milk (10 mL) were collected at entry (when the infant was four-weeks old). AF appears in both active and inactive forms [21], which was why it was purified by affinity chromatography for optimal binding sensitivity in the subsequent enzyme-linked immunosorbent assay (ELISA) [22]. In brief, 1 mL of breast milk or plasma was purified by running it through a column of Sepharose 6B CL (GE Healthcare BioSciences AB, Uppsala, Sweden), during which AF binds to the matrix by affinity binding. After washing with physiological phosphate buffered saline (PBS), AF was eluted with 1 mol∙L^-1^ 1 methyl-alfa-D-glucopyronoside in PBS. The eluate was kept at −20 °C until ELISA was performed. Ass. Prof Ewa Johansson and Prof Stefan Lange (Clinical microbiology, Sahlgrenska University Hospital, Gothenburg, Sweden) provided a monoclonal antibody of the IgM isotype (mAb43) to detect the active form of the protein [22]. All samples were run in duplicate and the mean was calculated. The concentrations of AF were expressed as equivalents against the reference peptide AF1–105, a peptide at the n-terminal part of AF including the active part of the full length protein.

Lactoferrin in breastmilk was analyzed using a Human Lactoferrin Elisa kit, HK329 (Hyocult, Biotec Inc., Uden, The Netherlands). The milk samples were diluted to 1.2 × 10^5^, run in duplicate, and the mean of each sample was calculated. 

The analyses of AF (purification and ELISA) and lactoferrin were performed by EG, who is an experienced biomedical analyst at the Biochemical laboratory at the Department of Odontology, Faculty of Medicine at Umeå University. 

Calcium in plasma was analyzed at the Department of Clinical Chemistry at Umeå University Hospital according to accredited methods. 

### 2.4. Samplings and Analysis of Oral Candida Colonization in Infants

The inside of the cheeks and tongue of the infants were sampled for Candida using a cotton swab. The samples were then cultivated on a selective media Oricult-n semi-quantitative dipslide (Orion Diagnostica, Espoo, Finland) and scored; Score 0 = no growth, Score 1 = 10^3^ CFU mL^−1^, Score 2 = 10^4^ CFU∙mL^−1^, Score 3 = 10^5^ CFU∙mL^−1^.

### 2.5. Data Analyzes

AF-levels in plasma and breast milk were analyzed descriptively in relation to each other and to the background factors in the mothers (i.e., age, parity BMI, mode of delivery, vaginal candida infection, and tobacco use) and infants (i.e., gestational age, birth weight, and sex). The distribution of levels of active AF in plasma and breast milk cannot be assumed to be normally distributed, since AF is predominantly present in the inactive form in healthy persons in a healthy environment [13] and the study cohort mainly consisted of healthy mothers. Therefore, we used non-parametric tests (Spearman’s correlation, Wilcoxon signed rank test), as well as both non-parametric and parametric tests after log transformation (Pearson’s correlation, Independent *t*-test, paired samples *t*-test). The log transformed values were used for the analytical analysis. A *p*-value of <0.05 was regarded as statistically significant. The Kolmogorov–Smirnov test was used to test for the normality distribution. Fisher’s exact test was used in the analysis when the numbers of observations were small. The software SPSS (version 24) was used in the analyses.

## 3. Results

### 3.1. Descriptive Results

The characteristics of mothers and infants at entry of the study are shown in Table 1. Fifty-four women were multipara, eleven infants were born by caesarean section, and four were born before gestational week 37. None of the women reported the consumption of an AF-inducing diet consisting of SPC-flakes^®^.

**Figure 1:**
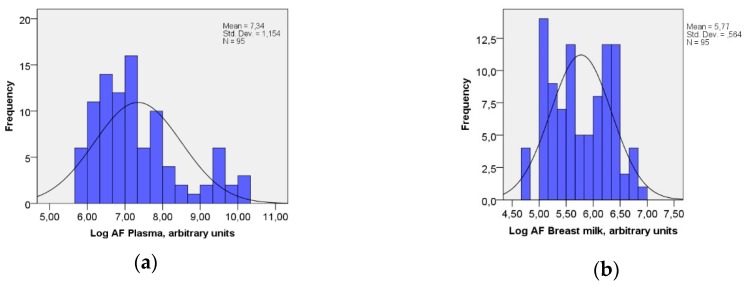
(**a**) Distribution of log AF-levels (arbitrary units) in the plasma Kolmogorov–Smirnov test of normality, *p* < 0.05. (**b**) Distribution of log AF-levels (arbitrary units) in breastmilk. Kolmogorov–Smirnov test of normality, *p* < 0.05.

Active AF was present in all samples of plasma and breast milk. The AF-levels were not normally distributed, neither in the plasma (Figure 1a) nor in breast milk (Figure 1b). Furthermore, there was a wider distribution of the level of active AF in the plasma than in the breast milk (Table 2). 

Figure 1a,b show the distribution of AF-levels (arbitrary units) in plasma and breastmilk.

### 3.2. Analytical Results

**Figure 2:**
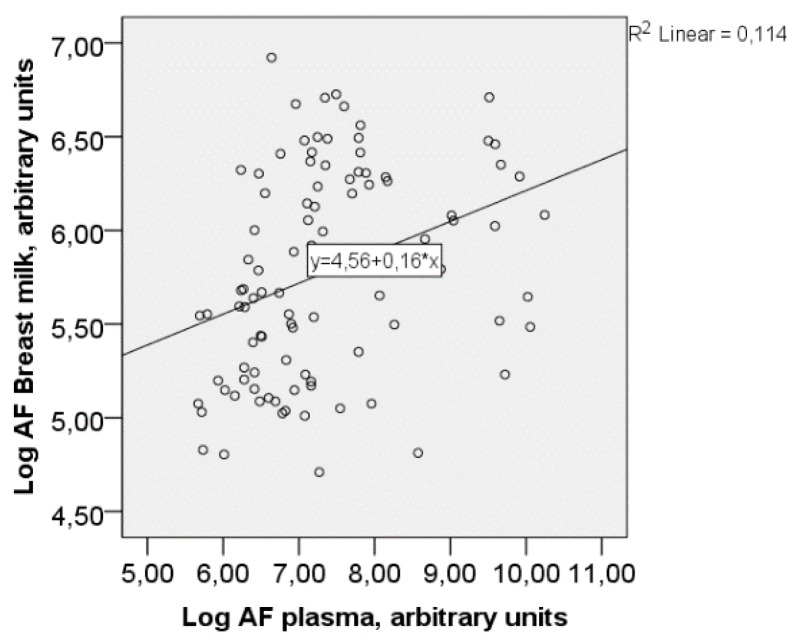
Correlation between the AF-levels (arbitrary units) in plasma and breast milk, Spearman’s rho = 0.403, *p* < 0.001.

There was a positive correlation between the levels of active AF in plasma and breast milk (Spearman’s rho = 0.403, *p* < 0.001) (Figure 2).

Figure 2 shows the correlation between AF-levels in the plasma and breast milk.

**Figure 3:**
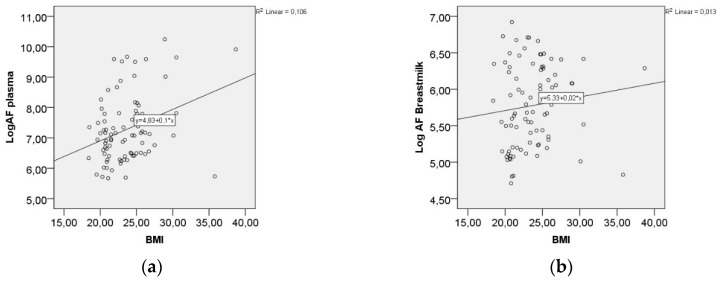
(**a**) AF-levels in plasma correlating to BMI, Spearman’s rho 0.252, *p* < 0.05; (**b**) AF-levels in breast milk in correlation to BMI, Spearman’s rho 0.193, *p* = 0.08.

We found that there was a positive correlation between AF in plasma and the BMI of the mother (Spearman’s rho 0.252, *p* < 0.05, Figure 3a), while the correlation to AF in breast milk was not statistically significant (Figure 3b). There were no differences in AF-levels depending on parity, gestational age, or mode of delivery. 

None of the mothers reported the intake of specially processed cereals (SPC-Flakes^®^), and we found no relation between AF and the intake of other cereals on the market (i.e., breakfast cereals, grain, porridge). Seventeen mothers reported a history of vaginal fungal infection during pregnancy and 14 of 17 received anti-fungal treatment. No correlation was found between the level of active AF in plasma or breast milk with neither vaginal fungal infection during pregnancy nor treatment. Furthermore, there was no correlation between infant Candida colonization and AF-levels in plasma or breastmilk (*p* > 0.05).

Twenty-five women reported having had sore nipples. There was a positive correlation between having had sore nipples and pain in the breast (Spearman’s rho 0.387, *p* < 0.01), inflammatory mastitis (Spearman’s rho 0.443, *p* < 0.01), and breast infection (Spearman’s rho 0.302, *p* < 0.01), but no correlation was demonstrated between having had sore nipples or pain in the breast and AF-level in plasma or breast milk.

Figure 3a,b show the AF-levels in plasma and breastmilk in correlation to maternal BMI.

**Figure 4:**
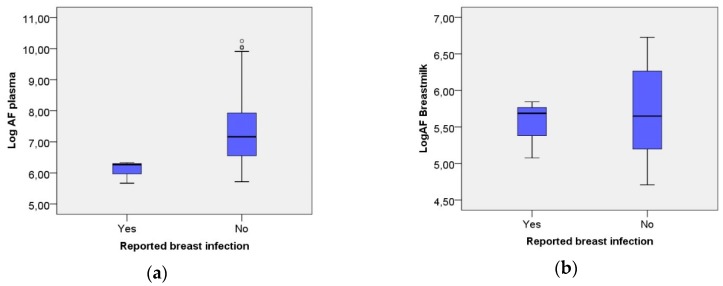
(**a**) Lower AF-levels in plasma related to having reported a breast infection (*n* = 3) compared with not reporting a breast infection (*p* < 0.05), unfilled circles indicate values 1.5 × *IQR* or more above the third quartile; (**b**) AF-levels in breast milk related to having reported breast infection (*n* = 3) compared with not reporting a breast infection (*p* > 0.05).

Three women reported having had a history of breast infection, and there was a correlation between breast-infection and low levels of active AF in plasma (Pearson’s correlation coefficient 0.220, *p* < 0.05 Pearson correlation), but not in breast milk (Pearson correlation coefficient 0.065, *p* > 0.05). In women who experienced a breast infection, the AF-levels in plasma were lower when compared to those without a history of infection (*p* < 0.05, independent samples *t*-test) (Figure 4a), while there was no difference in breast milk (*p* > 0.05) (Figure 4b). All three women with breast infections had AF-levels in plasma below the 25th percentile (*p* < 0.05, independent samples *t*-test). 

Figure 4a,b show the AF-levels and reported breast infection.

**Figure 5:**
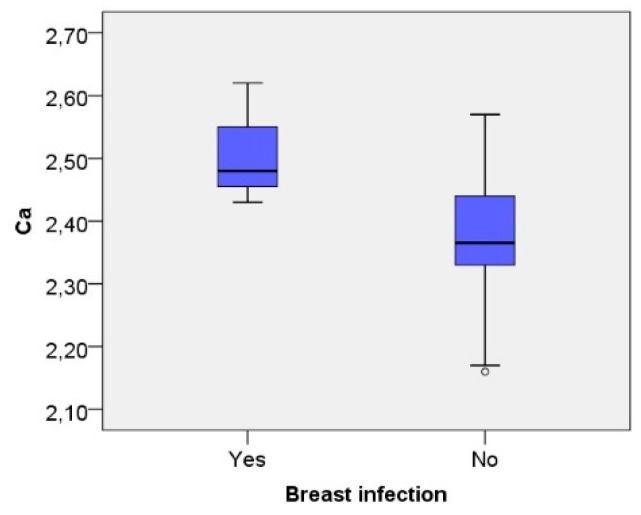
Calcium-levels related to reported breast infection, *t*-test, independent samples *p* < 0.01, unfilled circle indicates a value 1.5 × *IQR* or more below the third quartile.

There was a positive correlation between the calcium levels in plasma and having had a breast infection (Spearman’s rho—0.255, *p* = 0.05). Figure 5 displays the elevated calcium levels in mothers with a history of breast infection.

Furthermore, we found no association between reported breast complications and oral Candida colonization of the infant (Chi-Square test and Fischer exact test, *p* > 0.05) (Table 3).

Figure 5 shows the reported breast infection and calcium-levels.

**Figure 6:**
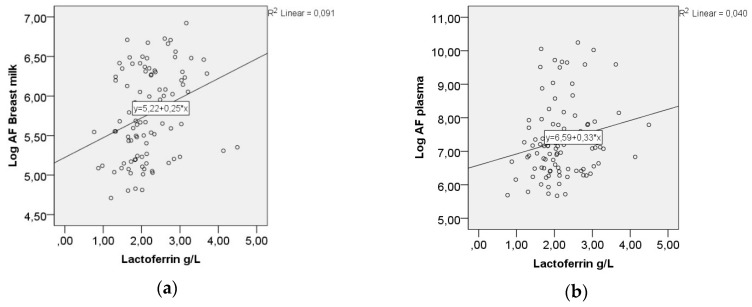
(**a**) Correlation between the AF-levels in breast milk and lactoferrin, Spearman’s rho 0.341, *p* < 0.01; (**b**) Correlation between the AF-levels in plasma and lactoferrin, Spearman’s rho 0.193, *p* = 0.06.

The mean lactoferrin level in breastmilk was 2.19 g/L, SD 0.68. A positive correlation was demonstrated between the AF-levels in breast milk and lactoferrin levels (Spearman’s rho 0.341, *p* < 0.01, Figure 6a). There was also a positive correlation between the AF-levels in plasma and lactoferrin; however, it did not reach statistical significance (Spearman’s rho 0.193, *p* = 0.06, Figure 6b).

Figure 6 shows the correlations between lactoferrin and AF-levels in breast milk and plasma.

## 4. Discussion

Active AF was present in all samples of plasma and breast milk from the mothers at one month post-partum. A positive relation was found between the levels of active AF in plasma and in breast milk, generally with lower levels in breast milk when compared to plasma.

In the majority of the mothers, the levels of active AF in plasma were low, possibly due to a generally healthy population with a high standard of living in an industrialized setting. We found a positive correlation between the BMI and level of AF in plasma. A higher BMI had previously been associated with an increased level of inflammation [23], and a higher AF-level might be a feed-back response to a higher degree of inflammation in general. However, we found no correlation between the AF-levels in breast milk and maternal BMI.

Furthermore, the levels of active AF in breast milk were also low in the majority of mothers. These results were consistent with the results from the intervention study by Svensson et al. [15]. The difference between the factors that correlated with the AF-levels in plasma and breast milk may suggest a local regulation of the AF-levels in the breast due to the health status of the breast and/or the infant. A relationship between the health status of both the mother and the infant and leucocyte count in breast milk has previously been described [24,25], where there were low baseline levels of leukocytes with increasing leukocyte levels, a proxy for the immune response, if the mother or infant had an infection. When the mother and infant are healthy, the origin of maternal cells in human milk is mainly from the mammary epithelium. However, during the first days postpartum and during periods of infection in either the mother or the infant, the human milk cells are dominated by immune cells from the maternal circulation [24,26,27]. 

The level of active AF was higher in the breast milk from mothers of infants colonized with Candida when compared to mothers of non-colonized infants, but the difference did not reach statistical significance [20]. Active AF in breast milk and lactoferrin were significantly correlated, as demonstrated by a weakly positive correlation between the AF in plasma and lactoferrin, but did not reach statistical significance. Lactoferrin has a role in the innate immunity. A study has shown enhanced levels of lactoferrin, expressed by endothelial cells and activated neutrophils, in breast milk from mothers breastfeeding sick infants [27]. The increased levels of AF in the present study did not protect the infant from Candida colonization [20], but the higher AF-level in breast milk may be due to the colonialization itself and might protect the infant and the mother from active disease.

Three women reported having experienced breast infection, and also had low AF-levels in plasma. The correlation between breast infection and low AF-levels in plasma may indicate an increased risk of breast infection in mothers with low AF-levels. However, there were only three mothers with a breast infection in our material, which is why larger and more detailed studies are needed before firm conclusions can be drawn, although our finding was consistent with the result from the intervention study by Svensson et al. [15]. Furthermore, in this study, we found no correlation between the oral Candida colonization of the infants and breast complications in the mother. The role of infant oral Candida colonization for maternal breast complications during lactation has previously been described with conflicting results [28,29], and there are ongoing studies [30].

Hence, AF is present in plasma and breast milk. Our hypothesis is that AF is involved in the immune defense and regulation of inflammation in the breastfeeding mother and her infant. The correlation between AF-levels in plasma and breast milk was positive and statistically significant. The pathways of the regulation of AF-levels between plasma and breast milk are yet to be fully understood. Is the level of active AF regulated by both the level in maternal plasma and by a local regulation in the breast due to the presence of colonization and/or infection in the breast or infant? Further studies are needed to determine the relation of AF in plasma and AF in breast milk, and the pathways of influence between them.

This study adds to the knowledge about AF in lactating mothers and how it associates with background factors, which has previously been very sparsely studied. The study was performed from 2011 to 2012, and the AF-levels demonstrated in it may be different in a different cohort or time period. However, in this cohort, the results demonstrated the basic levels of generally healthy woman in an industrialized setting. No consumption of specially processed cereals (SPC-flakes^®^) was reported and we found no relationship between the AF-levels and intake of other cereals on the market. 

The ELISA method we used was a non-commercial, in house version, which has now been further developed as a sandwich ELISA. Basic levels of AF may differ between individuals, and longitudinal studies with repeated measurements may show individual variability. Furthermore, the variability of AF in lactating mothers is unknown. Blood samples were only collected once in the study, at four weeks postpartum. Unfortunately, information on breast complications was not collected at the time of blood sampling, only at 12 months postpartum, and changes in AF-levels may have happened several times during this period of time. However, samples of plasma and samples of breast milk were collected at the same time, and breast complications most commonly appeared in the first weeks to months postpartum. The exact time for reported breast complications and relation in time to the collection of breast milk and plasma samples is unknown, which makes it difficult to establish firm conclusions about the causality between mastitis or sore nipples and AF-levels. Only three mothers in the cohort reported having had a breast infection, which is a low prevalence. However, all three mothers who experienced a breast infection during the first year after pregnancy had low AF-levels in plasma at one month post-partum. Furthermore, we found a positive correlation between calcium in plasma and having experienced a breast infection, which was consistent with the results from the study by Li et al. [31], who showed higher levels of calcium related to subclinical mastitis. They also demonstrated a positive correlation between calcium and the pro-inflammatory cytokine IL-6. Calcium is known to be involved in the recruitment of neutrophils to sites of inflammation. Changes in intracellular calcium levels play an important role in neutrophil activation and function [32].

## 5. Conclusions

Active AF was present in plasma and breast milk in all mothers and their levels were positively correlated. Active AF was predominantly low in both plasma and breast milk, but there was a broader distribution of AF-levels in plasma when compared with the AF-levels in breast milk. The results suggest a possible local regulation of AF-levels in the breast, as well as a co-regulation with AF-levels in plasma. This study contributes to the knowledge regarding the basic patterns of active AF in plasma and breastmilk. The study was explorative and further longitudinal studies on active AF in breast milk and plasma are needed.

## Figures and Tables

**Table 1:** Maternal and infant characteristics.

Factor	Total
	*N* = 95
**Maternal characteristics**	
Age (years) Mean (SD) (range)	32 (4.5) 21–44
Primiparous, *n*	44
BMI* (kg/m^2^) Median (range)	23 (19.6–30.4)
Cesarean Section, *n*	9
Vaginal Candida Infection, *n*	16
Treatment for Candida Infection, *n*	15
Tobacco Use	
Maternal Smoking, *n*	2
Paternal Smoking, *n*	1
Maternal Use of Snuff, *n*	2
**Infant characteristics**	
Gestational Age < 37 Weeks	3
Birth Weight, Gram Mean (SD)	3552 (481.5)
Female *n*	50
Oral Candida, *n*	11
**Breast complications**	
Pain in the Breast, *n*	19
Sore Nipples, *n*	25
Mastitis, *n*	17
Infection, *n*	3

* BMI data were collected from 84 of the mothers.

**Table 2:** Distribution of AF (arbitrary units) in plasma and breast milk, before and after log-transformation.

AF Plasma Median (range)	1240 (27,835)
log AF Plasma, Mean (SD)	7.34 (1.15)
AF Breast Milk, Median (range)	293 (904)
log AF Breast Milk, Mean (SD)	5.77 (0.56)

**Table 3:** Maternal breast complication and infant oral Candida colonization.

Maternal Breast Complication		Infant Oral Candida Colonization		
		No Candida	Candida	*p*-Value
		*N*	*N*	
Mastitis	yes	17	0	>0.05
	no	56	9	
Breast Infection	yes	3	0	>0.05
	no	69	9	
Sore Nipples	yes	23	2	>0.05
	no	50	6	
Pain in Breast/Nipples	yes	17	2	>0.05
	no	56	7	
